# Study on Alkali-Activated Slag Mortar Based on Co-Modified Recycled Fine Aggregate with Nano-SiO_2_ and Sodium Silicate Integrating Waste Liquid Recycling

**DOI:** 10.3390/ma18214889

**Published:** 2025-10-25

**Authors:** Qiushi Su, Changbai Wang, Jimin Liu, Qinghua Liu

**Affiliations:** 1 School of Civil Engineering and Architecture, Anhui University of Science and Technology, Huainan 232001, China; sqs1216469248@163.com (Q.S.); jimliu@aust.edu.cn (J.L.); 18831788027@163.com (Q.L.); 2 State Key Laboratory of Safe Mining of Deep Coal and Environmental Protection, Anhui University of Science and Technology, Huainan 232001, China

**Keywords:** alkali-activated slag mortar, recycled fine aggregate, co-modification, waste liquid recycling, sustainability

## Abstract

The widespread use of recycled fine aggregate (RFA) is hindered by its porous and weak adhered mortar. In this study, a nano-SiO_2_–sodium silicate mixed solution (NMS) was used to soak and strengthen the adhered mortar. Alkali-activated slag was adopted as the cementitious material, and the resulting treated waste liquid (RNMS) was recycled as a sodium silicate source for the alkali activator. The effects of modified RFA (MRFA) incorporation and RNMS use on the performance, economic, and environmental benefits of alkali-activated slag recycled fine aggregate mortar (AASRM) were evaluated. Compared with the control group, mortars using only MRFA showed significantly improved performance, with a 28-day compressive strength increase of 57.6% (reaching 38.3 MPa) and enhanced workability. The capillary water absorption and 90-day drying shrinkage rates decreased by 49.5% and 40.2%, respectively. Microstructural analysis revealed that NMS treatment promoted the formation of additional C-(N)-A-S-H gel, thereby densifying the surface of the RFA and strengthening the interfacial transition zone (ITZ). More importantly, using RNMS as the alkali activator source maintained the excellent performance of the AASRM mortar, with the compressive strength reaching 95.6% of that prepared with a fresh alkali activator, while effectively reducing material costs and embodied carbon. This study not only successfully applies MRFA in alkali-activated mortar systems but also provides an effective approach for the in situ recycling of treated waste liquid.

## 1. Introduction

Accelerated global urbanization and burgeoning infrastructure development have resulted in the generation of substantial quantities of construction and demolition waste (C&DW) [1], within which discarded concrete constitutes a significant proportion of the waste [2]. Modern concrete production relies heavily on the consumption of natural aggregates (NAs), such as sand and gravel, which account for approximately 75% of the total volume [3]. However, the extraction of these virgin resources leads to considerable ecological degradation [4]. Consequently, the valorization of waste concrete through its conversion into recycled aggregates (RAs) is of paramount importance for mitigating the depletion of natural resources and alleviating environmental pollution [5,6]. Following processes such as crushing and sieving, waste concrete is transformed into recycled coarse aggregate (particle size > 4.75 mm) and recycled fine aggregate (RFA, particle size ≤ 4.75 mm), in accordance with Chinese standards GB/T 25176 and GB/T 25177 [7,8,9]. The feasibility of utilizing recycled coarse aggregate for industrial-scale production and its potential to enhance circularity in the construction sector have been extensively validated in previous studies [6,10,11]. In contrast, the application of RFA remains comparatively underexplored. Originating from the comminution of hardened concrete, RFA is distinct from natural sand and is characterized by an irregular morphology, high angularity, and rough surface texture [12,13]. Furthermore, its surface is encapsulated by a layer of adhered old cement mortar [14], which is inherently porous and weak. This residual mortar is the primary contributor to the elevated water absorption and porosity observed in the RFA [15,16]. These inferior inherent properties ultimately compromise the mechanical performance and durability of mortars incorporating RFA. Addressing these deficiencies through effective pretreatment strategies is crucial for enhancing the quality of this secondary raw material.

Currently, owing to their ultra-high specific surface area and reactivity, nanomaterials have been introduced into concrete technology [17,18]. Their application has been demonstrated to effectively enhance the mechanical properties, durability, and microstructure of mortar and concrete [19,20,21,22]. Among these materials, which are typically defined by a particle size range of 1–100 nm [23], nano-silica (NS) has been extensively utilized to improve the performance of recycled aggregate composites. This widespread adoption is attributable not only to its relative cost-effectiveness but also to its multifaceted contributions, including nucleation, micro-filling, and pozzolanic effects, which are conducive to microstructural refinement [24,25,26]. Concurrently, the consumption of structurally weak and porous calcium hydroxide (CH) is facilitated by NS, leading to the formation of additional calcium–silicate–hydrate (C-S-H) gels. This resultant gel, possessing a denser structure and higher intrinsic strength, strengthens the composite material [27,28]. The benefits of NS modification have been substantiated in numerous studies. Li et al. [29] employed three distinct NS treatment methods and reported an increase in the microhardness of both the adhered old mortar and the new mortar matrix proximate to the interfacial transition zone (ITZ), which culminated in an overall enhancement of concrete performance. Chen et al. [30] identified an optimal concentration of 2 wt% for strengthening RA via immersion in an NS solution and attributed the improvement in the RA’s pore structure to the pozzolanic and filling effects of NS. Zeng et al. [31] similarly achieved enhanced mechanical properties and steel corrosion protection in recycled aggregate concrete by modifying RA through a one-hour immersion in an NS suspension. Subsequent studies have explored the synergistic effects of multiple modifying agents. Kumar et al. [32] utilized a combination of silica fume and sodium silicate to treat RA, resulting in the successful filling of cracks and interconnected pores within the aggregates. Liu et al. [33] reported the superior efficacy of a composite solution (2 wt% NS and 15 wt% sodium silicate) over single-solution treatments, achieving significant reductions in the crushing value and water absorption of RA alongside an increase in its apparent density. Liu et al. [34] further investigated the synergistic modification mechanism of a nano-silica–sodium silicate mixed solution (NMS) and revealed that both components participate in secondary hydration reactions to form C-S-H gel, thereby densifying the ITZ microstructure. Despite these advancements, a notable drawback persists: the residual NMS solution after the treatment contains unreacted sodium silicate and NS. Its direct disposal would result in resource wastage and potential environmental contamination. Furthermore, the conventional reliance on Ordinary Portland Cement (OPC) as the primary cementitious material is associated with substantial energy consumption and carbon dioxide emissions, a practice that runs counter to the prevailing global imperative for green, low-carbon development [35,36].

Alkali-activated materials (AAMs) have emerged as a promising and environmentally friendly alternative to OPC in concrete production [37,38]. Among the various precursors utilized for AAMs, ground granulated blast-furnace slag (GGBS) is the most prevalent [39]. The activation of latent hydraulic activity within the aluminosilicate structure is typically achieved using a strong alkaline solution, such as an alkali–metal hydroxide. This process leads to the formation of a primary binding phase, calcium–(sodium)–aluminate–silicate–hydrate (C-(N)-A-S-H) gel, which imparts superior properties to the material compared to traditional cement-based composites [40,41]. However, the widespread application of AAMs poses challenges. The rheological behavior of AAMs differs from that of OPC. Specifically, the sodium content in the alkaline solution can significantly alter the viscosity of the mortar in its fresh state. The use of solutions with high molar concentrations may result in a loss of workability [42,43]. Simultaneously, AAMs exhibit a notable deficiency in volume stability, manifesting as significantly higher drying shrinkage relative to OPC-based systems [44]. This excessive shrinkage can induce microcracking and compromise the structural integrity of the material. Some studies have suggested that the finer pore structure inherent in AAMs is responsible for this phenomenon, as it leads to elevated capillary stresses and, consequently, greater shrinkage [45,46]. To date, a combination of sodium silicate (waterglass) and sodium hydroxide (NaOH) solutions is widely recognized as one of the most effective and frequently cited alkaline activator systems [47]. However, the use of these commercial alkalis will undoubtedly increase production costs and have an adverse environmental impact. Therefore, seeking and utilizing suitable waste alkalis to replace commercial activators is an important step toward the truly eco-friendly design of building materials [48]. Notably, the NMS waste liquid produced after pretreating RA, as mentioned above, contains unreacted sodium silicate and NS components. By using the waste alkaline solution treated through this in situ recycling method as a raw material for alkali activators, pollution caused by the discharge of treated waste liquid can be eliminated, material costs can be reduced, and thus the sustainability and economic benefits of building materials can be enhanced.

In this study, recycled fine aggregate (RFA) modified with a nano-SiO_2_-sodium silicate mixed solution (NMS) was applied to a more sustainable alkali-activated material (AAM) system compared to OPC. The effects of different modified RFA (MRFA) incorporation levels on the mechanical and durability properties of alkali-activated slag mortar with recycled fine aggregates (AASRMs) were analyzed. Simultaneously, the feasibility of using recycled NMS (RNMS) as a raw material for alkali activators was explored to enhance environmental and economic benefits. The experimental groups included a blank group with unmodified RFA (NM), MR groups with different MRFA incorporation levels, and RMR groups with different MRFA incorporation levels and RNMS. Tests included flowability, capillary water absorption, compressive strength, and drying shrinkage. Scanning Electron Microscopy (SEM) was used for microstructural characterization to observe the surface morphology of the MRFA and the microstructure of the interfacial transition zone (ITZ). X-ray diffraction (XRD) was used for phase analysis of hydration products. In addition, analyses of economic and environmental benefits were conducted. This study explores the use of MRFA in alkali-activated systems and pioneers a practical solution for recycling waste alkali solutions, thus achieving environmentally friendly and sustainable production.

## 2. Materials and Methods

### 2.1. Materials

**Figure 1:**
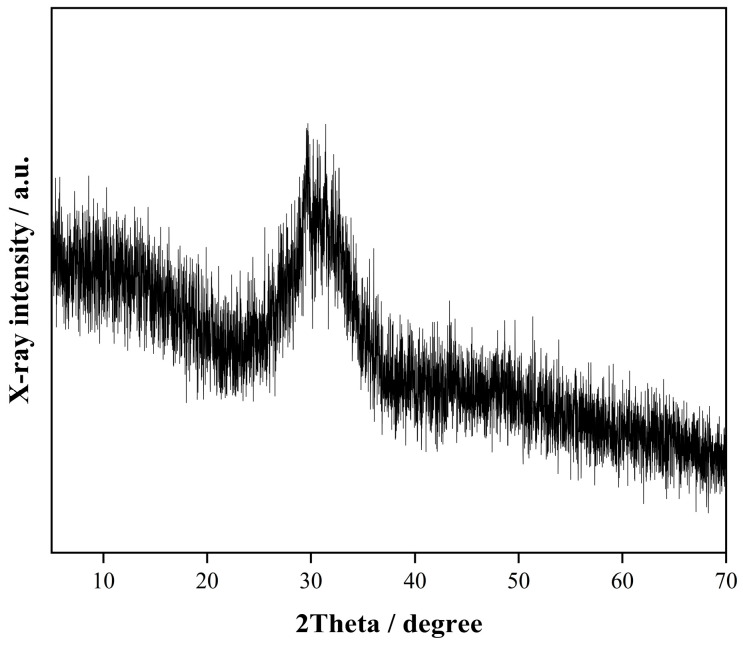
XRD pattern of GGBS.

**Figure 2:**
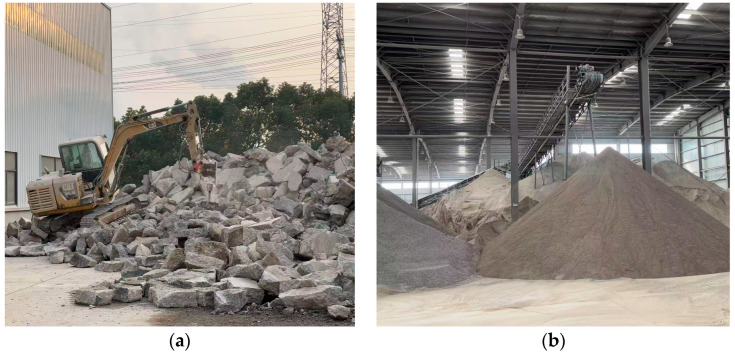
Waste concrete recycling process at Huainan Yishan New Building Materials Technology Co., Ltd., in Huainan, Anhui, China: (**a**) crushing and recycling waste concrete, (**b**) sorting recycled concrete aggregates.

Ground granulated blast-furnace slag (GGBS) with an apparent density of 2800 kg/m^3^ was sourced from Fuyang Xinyuan Building Materials Co., Ltd., located in Fuyang, Anhui, China. The chemical composition determined by X-ray fluorescence (XRF) 1000 spectrometry is shown in Table 1, while its X-ray diffraction (XRD) pattern is shown in Figure 1. The RFA used in this study originated from crushed C40 waste concrete recovered by Huainan Yishan New Building Materials Technology Co., Ltd., in Huainan, Anhui, China. The fineness modulus was 2.69. Figure 2a,b illustrate the factory waste concrete recycling process, which typically includes separation, crushing, washing, and screening to achieve the required aggregate size. The sodium silicate solution was produced by Henan Luboshi New Materials Technology Co., Ltd., in Zhengzhou, Henan, China. It had a modulus of 3.26 and contained 26.2% SiO_2_, 8.3% Na_2_O, and 65.5% H_2_O. Analytical grade sodium hydroxide (NaOH) pellets of 96% purity were procured from Xilong Scientific Co., Ltd., in Shenzhen, Guangdong, China. Hydrophilic NS, with 99.5% purity and an average particle size of 15 nm, was acquired from Shanghai Macklin Biochemical Technology Co., Ltd., in Shanghai, China. All mixtures were prepared using urban tap water.

### 2.2. Methodology

#### 2.2.1. Modification Process of RFA and Waste NMS Recycling Process

**Figure 3:**
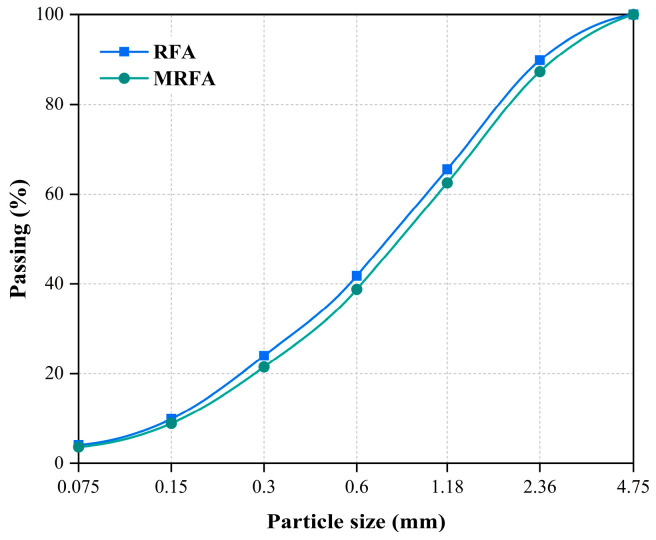
Particle size distribution curves of RFA and MRFA.

**Figure 4:**
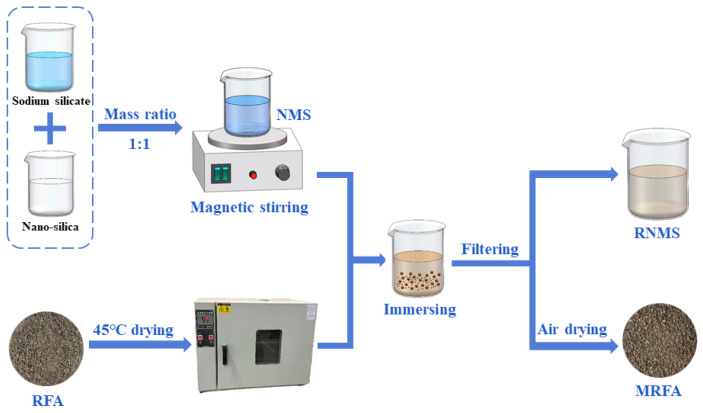
Schematic view of NMS modification and RNMS recycling.

The nano-silica–sodium silicate mixed solution (NMS), utilized for RFA property enhancement, was formulated by combining 2 wt% NS solution and 15 wt% sodium silicate solution at a 1:1 mass ratio. This specific formulation is based on the systematic studies by Liu et al. [33,34], whose research demonstrated that this combination provides optimal synergistic effects in the modification of recycled aggregates. Their findings indicated that a 15 wt.% sodium silicate solution formed the primary blocking gel layer, while a 2 wt.% NS solution was used to fill the nanopores and promote the nucleation of hydration products, thereby creating a dense microstructure. Using this validated formulation, the present study ensured effective aggregate modification, laying a solid foundation for evaluating its performance in alkali-activated slag systems. This mixture was subsequently subjected to magnetic agitation for 6 h to ensure uniform dispersion of the nano-silica [34]. Prior to modification, the RFA was dried in an oven at 45 °C until a constant weight was attained, which was defined as a mass variation not exceeding 1% over a 2-h period. Choosing this mild drying temperature is aimed at accelerating moisture removal while avoiding thermal damage or microcracks in the attached mortar. After cooling to ambient temperature, the RFA was immersed in the prepared NMS for 24 h. A 1:1 mass ratio of RFA to NMS was maintained to ensure that the aggregate was completely immersed throughout the entire process and that the concentration of the modifier in the solution remained stable. Excess NMS was removed by filtration through a 200-mesh sieve, and the filtrate was stored for subsequent use. The material retained on the sieve, designated as modified RFA (MRFA), was air-dried to remove residual moisture. The physical properties of both RFA and MRFA are listed in Table 2. The particle size distribution curves of RFA and MRFA are presented in Figure 3. The recovered filtrate, termed recycled NMS (RNMS), was allowed to settle for 24 h, after which the supernatant was collected. Owing to the underutilized sodium silicate and sufficient NS content, this solution was deemed a viable raw material for the alkaline activator. The process of RFA modification with NMS and subsequent RNMS recovery is schematically illustrated in Figure 4.

#### 2.2.2. Mix Proportion and Sample Preparation

The mix proportions of the mortars were designed in accordance with the Chinese standard “Specification for Mix Proportion Design of Masonry Mortar” (JGJ/T 98–2010) [49], targeting an M15 strength grade, which corresponds to a 28-day compressive strength of 15 MPa. The base formulation per cubic meter of mortar comprised 312 kg of GGBS, 1320 kg of recycled fine aggregate, and a total water content of 280 kg. The total water usage is the sum of the water contained in the sodium silicate solution or RNMS and the amount of additional water added. The alkaline activator was formulated to have a modulus (Ms, molar ratio of SiO_2_ to Na_2_O) of 1.0 and an alkali equivalent (mass ratio of Na_2_O to binder) of 6%. This was achieved by blending a sodium silicate solution (Ms = 3.26) with 96% purity NaOH pellets and supplementary water. The choice of a 6% alkali dosage is because this concentration is sufficient to effectively dissolve the slag precursor and initiate subsequent polycondensation reactions, thereby ensuring robust mechanical strength development [50]. Lower alkali concentrations may lead to incomplete activation and insufficient strength, whereas excessively high concentrations can cause premature shrinkage, cracking, and increased brittleness of the mortar [51]. In addition, selecting Ms = 1.0 aimed to achieve an ideal balance between workability and mechanical performance. As demonstrated by Burciaga-Díaz et al. [52], a modulus that is too high (such as 1.2 or above) can adversely affect the setting and strength growth due to an excessive increase in the activator’s viscosity. Conversely, a modulus that is too low lacks the reactive silicate monomers necessary for the rapid formation of a robust C-(N)-S-H network structure. This value has also been successfully applied in recent geopolymer studies by Liu et al. [53], ensuring good workability while promoting the development of a dense and high-strength cementitious microstructure. For the activators prepared using the recycled solution, the potential losses of sodium silicate within the RNMS during the recovery process were disregarded in the formulation. The experimental program comprised nine distinct mortar mixture types. These were divided into two main categories based on the source of the activator. The first category, which utilized an alkaline activator prepared from fresh reagents, included the control mixture (NM) and MR series. The NM control was formulated with 100% unmodified RFA, whereas the MR series involved replacing the RFA with MRFA at mass fractions of 10%, 30%, 50%, and 100% (denoted as MR-1, MR-3, MR-5, and MR-10, respectively). The second category, the RMR series, was specifically designed to evaluate the recycled activator used. It mirrored the MRFA replacement levels of the MR series but was formulated using recovered RNMS as the sodium silicate source. The detailed mix proportions for all series are presented in Table 3.

### 2.3. Test Methods for Mortar Properties

#### 2.3.1. Fluidity Test

The fluidity of the AASRMs was determined by measuring the spread diameter of each paste group under specified vibration conditions, in accordance with the “Test method for fluidity of cement mortar” (GB/T 2419-2005) [54]. Following mixing, the paste was rapidly introduced into the test mold in two separate layers. Subsequent tamping was performed using a tamping rod, as stipulated by the standard. Following the removal of the mold collar, any paste extending above the mold was scraped off at a horizontal angle using a knife. The conical round mold was then gently lifted vertically. The activation of the flow table was immediate, and twenty-five jolts were completed within 25 ± 1 s at a frequency of one jolt per second. The fluidity of the mortar was reported as the average of two diametrical measurements taken in mutually perpendicular directions on the bottom surface of the spread mortar. This value was rounded to the nearest integer, with the unit being mm. For each mortar mix ratio, the entire testing process was repeated for three independent batches. The final average flowability and standard error were obtained from three parallel measurements, with error bars indicating the standard deviation.

#### 2.3.2. XRD Test

X-ray diffraction (XRD) testing was performed on the powder samples of all the specimens to determine their mineralogical composition. The scanning speed was set to 5°/min with a scanning range of 5–80°. Before testing, the mortar samples were dried in an oven at 40 °C to stop hydration. Specifically, mortar samples were obtained by crushing the central region of the 28-day compressive strength cube specimens. The samples to be tested were ground into powder and passed through a 200-mesh square sieve.

#### 2.3.3. Capillary Water Absorption Test

The capillary water absorption rate of the sample (φ100 mm × 50 mm) was determined and analyzed according to ASTM C1585 [55]. Mortar samples, which were standardized and cured for 28 d, were dried to a constant weight at 40 °C and then coated with epoxy resin on all sides except one. During testing, the top surface of the specimen was sealed with a plastic cover, whereas the bottom surface was immersed 1–3 mm in deionized water. The sample is brought into contact with water and removed from the water at the time intervals specified in the standard. Any free water on the bottom of the sample was wiped off with a damp paper towel, and the sample was weighed and placed back in position, and the data were recorded. The average value was used as the water absorption rate of the sample. The weight of the specimen was measured at specified intervals, and the capillary absorption rate was calculated using Equation (1). Three cylindrical specimens were tested for each mortar mix ratio. The average capillary water absorption rate and standard error were obtained from three parallel measurements, with the standard deviation represented by the error bars.

(1) $$I=\frac{m_{t}}{\pi r^{2}}$$

where *I* is the capillary water absorption (mm), *m_t_* is the mass increase in the sample at time *t*, and *r* is the radius of the cylindrical specimen (50 mm).

#### 2.3.4. Compressive Strength Test

According to the Chinese standard “Standard for Test Method of Performance on Building Mortar” (JGJ/T 70-2009) [56], the compressive strength of 70.7 mm × 70.7 mm × 70.7 mm cubic specimens was evaluated. Fresh mortar was poured into the molds and left to stand at room temperature (20 ± 5 °C) for 24 ± 2 h, after which the specimens were numbered and demolded. Immediately after demolding, the specimens were placed in a standard curing chamber at a temperature of 20 ± 2 °C and relative humidity above 90% to produce hardened cubes for 3, 7, and 28 days. The average compressive strength and standard error were obtained from three parallel measurements, with the standard deviation represented by error bars.

#### 2.3.5. Drying Shrinkage Test

According to the “Standard for Test Method of Performance on Building Mortar” (JGJ/T70-2009) [56], measurements were carried out using samples measuring 40 mm × 40 mm × 160 mm. The test specimens were prepared and demolded according to the standard, with three samples in each group. The samples were first cured in water at 20 ± 2 °C for 7 days, then removed, and their surfaces were dried with a towel. Subsequently, they are transferred to a curing chamber maintained at 20 ± 2 °C and a relative humidity of 60% ± 5%. The length of the samples when they were first placed in the curing chamber was recorded as the initial length. A length comparator and displacement sensor were used to measure the change in length of the mortar samples at specified intervals of up to 90 d. Drying shrinkage at each specified age was defined as the change in length divided by the initial length of the sample. For each mortar mix ratio, the drying shrinkage strain was the average value from three replicate specimens, and the error bars in the corresponding figures represent the standard deviation.

#### 2.3.6. Microstructure Characterization

The microstructures of the AASRMs samples aged 28 d were observed using a ZEISS supra 55 scanning electron microscope (SEM) (Carl Zeiss, Oberkochen, Germany). To halt further hydration, samples from the central region of the compressive strength cubes were collected and soaked in isopropanol for over a week. Owing to the poor conductivity of the mortar samples, the vacuum-dried specimens were coated with gold particles for 180 s to achieve high conductivity. To provide direct chemical evidence for the observed phases, we conducted energy-dispersive X-ray spectroscopy (EDS) analyses of representative areas.

## 3. Results and Discussions

### 3.1. Fluidity of Mortar

**Figure 5:**
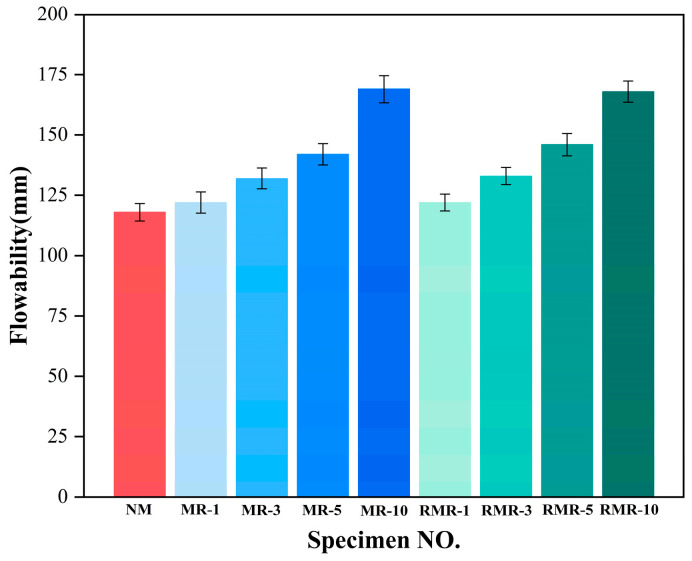
Fluidity of 28 d AASRM pastes.

Figure 5 shows the fluidity of the AASRMs. The fluidity increased significantly with increasing MRFA content, and the fluidity of the MRs and RMRs was essentially the same for the same incorporation. As shown in Figure 5, the fluidity of the fresh mixture without MRFA was 118 mm. Compared with the blank group NM, the fluidity of the MRs increased by 3.4%, 11.9%, 20.3%, and 43.2%, whereas that of the RMRs increased by 3.4%, 12.7%, 22%, and 42.4%, respectively. The fluidity of the AASRM decreased as the RFA content increased. Li et al. [57] reached a similar conclusion in their experiments. The high water absorption rate of RFA results in the absorption of a certain amount of free water during the mixing process. Additionally, the uneven edges and rough surface of RFA increase the friction during the flow of AASRMs, thereby reducing the fluidity [58,59]. Simultaneously, the presence of fine particles in RFA increases the viscosity of the paste, which in turn hinders its flow [60]. However, by soaking MRFA in NMS for modification, the water absorption rate is reduced, and the loose fine particles became encapsulated by a gel film on the surface of the aggregate, forming a dense protective shell after air-drying. This type of modification reduces the absorption of water from the paste, lowers internal friction, and improves the flowability of AASRMs.

### 3.2. XRD Analysis

**Figure 6:**
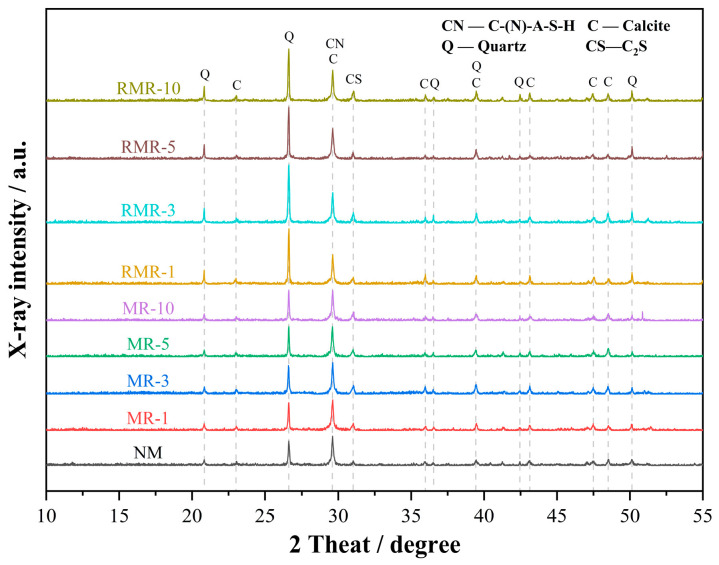
XRD patterns of 28 d AASRM pastes.

Figure 6 shows the XRD patterns of the ground AASRM specimens after 28 d of curing. According to the JCPDS and relevant literature, prominent diffraction peaks of quartz present in all samples were identified, originating from the RFA and MRFA used in the AASRMs. In addition to quartz, the main hydration products include C-(N)-A-S-H gel, calcite, and dicalcium silicate. The dicalcium silicate peak at 2θ = 31.03° mainly originated from unhydrated particles in the GGBS precursor. The calcite peak at 2θ = 29.41° partially overlaps with the characteristic amorphous hump of the C-(N)-A-S-H gel, which may be due to minor carbonation during mixing, sample preparation or testing. Among these phases, the C-(N)-A-S-H gel, an inorganic polymer formed during the alkali-activation reaction, significantly contributes to the strength of AASRMs. The hydration products of the NM, MR, and RMR groups were consistent, indicating that neither the in-corporation of MRFA nor the use of RNMS as the source of the alkali activator significantly changed the main reaction products of AASRMs.

Notably, the RMRs exhibit higher quartz peaks at 2θ = 20.86°, 26.64°, and 50.14° compared to the NM and MRs, implying that the residual amorphous silica in the RNMS is not fully utilized. This can slightly increase the modulus of the alkali activator prepared with RNMS, thereby lowering the pH of the silicate species in the alkaline solution, increasing the degree of polymerization of the silicate species, and reducing the reactivity of the alkaline solution [61]. Nevertheless, the heights of the C-(N)-A-S-H peaks at 2θ = 29.61° and 31.03° in the RMRs are consistent with those of the other groups, indicating that, despite these differences, alkali activators prepared using RNMS as a raw material can still effectively promote the alkali-activation reaction and generate sufficient C-(N)-A-S-H gel.

### 3.3. Capillary Water Absorption

**Figure 7:**
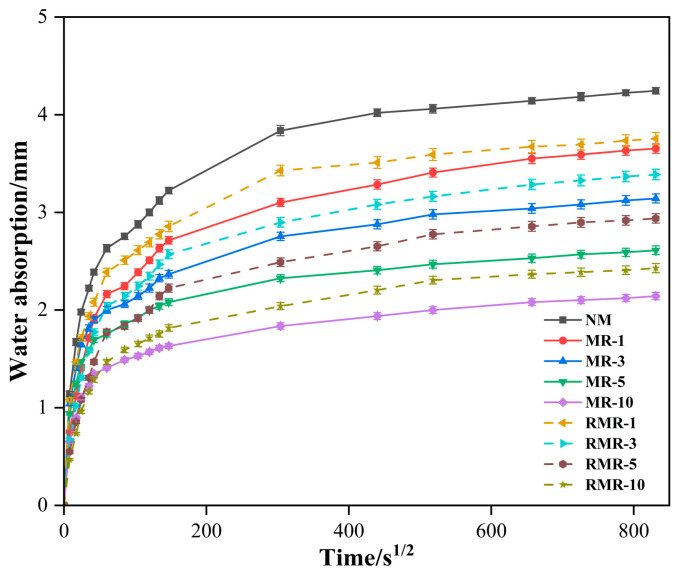
Capillary water absorption of mortars at 28 d.

Figure 7 shows the capillary water absorption rates of the AASRMs at 28 d. The capillary pores on the surface of the NM sample rapidly absorbed water owing to surface tension within 1 h, reaching 62% of the maximum absorption height. Subsequently, the absorption rate gradually decreased until it saturated at approximately 6 d, with a final water absorption height of 4.245 mm. This is consistent with the findings of Gao et al. [62], who reported that the highest RA content was accompanied by the highest capillary water absorption rate. This can be attributed to the high porosity, weak interfacial transition zone (ITZ), and low density characteristics of the RFA [63].

However, after partially incorporating MRFA modified by soaking in NMS for 24 h and air-drying, the capillary water absorption height of the MRs was significantly reduced. As the proportion of MRFA increased, the capillary absorption height of MRs decreased by 13.9–49.5%, and the final water absorption height of MR-10 was 2.143 mm. This indicates that, owing to the synergistic effects of NS physically filling microcracks and pores, as well as film formation on the surface by sodium silicate, the aggregate performance is enhanced, and the water absorption rate of MRFA is reduced. Meanwhile, the residual NS and sodium silicate particles on the surface can also participate in alkali-activated reactions with the matrix [64], promoting the formation of more hydration products at the interface, thereby strengthening the ITZ between the aggregate and matrix. The ITZ is a narrow region where the cement paste bonds to the aggregate, and previous studies have identified it as a key factor in determining the mechanical and durability properties of concrete/mortar [65,66,67]. By improving the structure of the ITZ, the capillary absorption height of the MRs was reduced, and the microstructure of the AASRMs was densified.

When RNMS was used as an alkali-activated raw material to prepare RMRs, a development pattern similar to that of MRs was observed; that is, as the replacement rate of MRFA increased, the capillary water absorption height of RMRs was significantly reduced by 11.5–42.8% compared to NM, with the water absorption height of RMR-10 being 2.429 mm. Notably, compared with MRs at the same replacement rate, the capillary water absorption height only slightly increased by 2.8–13.3%. This indicates that RMRs also possess a relatively dense microstructure, confirming the feasibility of using RNMS as a raw material for alkali activation.

### 3.4. Compressive Strength

**Figure 8:**
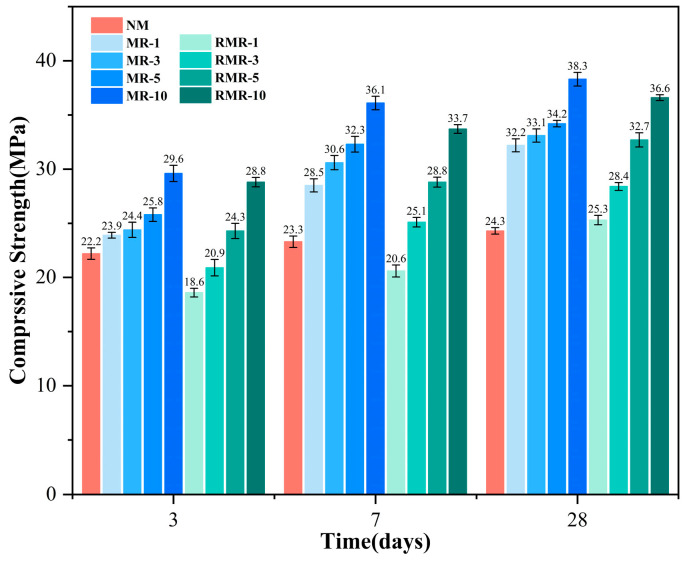
Compressive strength of mortars.

Figure 8 shows the compressive strength of AASRMs. The compressive strength of the control group NM had already developed in the early stage, with a strength of 24.3 MPa at 28 d, which is only a 9.5% increase compared to that at 3 d, making it the lowest among the AASRM mortars. This result is consistent with the findings of Xie et al. [68], showing that the compressive strength of alkali-activated mortar significantly decreases when untreated RFA completely replaces natural aggregates, decreasing from 50.4 MPa to 19.8 MPa, a reduction rate of 60.71%. Because GGBS was used as the precursor for alkali activation and both the modulus and alkali equivalent of the alkali activator were kept essentially the same, the primary source of strength loss was RFA. The high water absorption of RFA reduces the effective water-to-binder ratio in the paste [69] and alters the local moisture distribution within the new and old mortar matrix ITZ, thereby weakening the efficiency of matrix hydration reactions. This may adversely affect the formation of new ITZ structures, both between the original aggregate and the new matrix, and between the adhered mortar and the new matrix. Meanwhile, the inherent porosity and microcracks in the attached old mortar further weaken the ITZ [70], negatively affecting the mechanical properties of the AASRMs.

Compared to the blank NM group, the compressive strength of the MRs developed rapidly in the early stage and continued to grow steadily in the later stage. This rapid development of strength is particularly remarkable when compared with other modification strategies for traditional OPC systems. For example, Gomes et al. [71] re-ported that even with a 100% replacement of RFA optimized by carbonation in OPC mor-tar, the 7-day and 28-day compressive strengths were only 28.3 MPa and 35.2 MPa, respectively. In stark contrast, the MR-10 mortar in this study achieved a 7-day compressive strength as high as 36.1 MPa, surpassing the final strength of the carbonated mortar in the Gomes study and reaching 94.3% of its own 28-day strength. This clearly demonstrates that the combination of NMS modification technology and the inherent early strength feature of alkali-activated systems can achieve superior strength development compared with traditional OPC systems. Simultaneously, the 3-day compressive strength had al-ready increased by 7.7–33.3%, and the 28-day compressive strength further expanded the growth margin to 32.5–57.6%. The 3-day compressive strength had already increased by 7.7–33.3%, and the 28-day compressive strength further expanded the growth margin to 32.5–57.6%. It can also be observed that as the MRFA replacement rate increases, the compressive strength of the MRs also improves. When MRFA was used as the sole aggregate, the 28-day compressive strength of the prepared mortar sample reached as high as 38.3 MPa, which was 18.9% higher than that of MR-1. This indicates that after modification by NMS soaking, sodium silicate bonds and permeates onto the pores and microcracks of the RFA surfaces, while NS acts as a filler and reacts with Ca(OH)_2_ through a pozzolanic reaction to form additional C-(N)-A-S-H gel. This synergistic effect seals the pores and microcracks on the RFA surface, resulting in a denser microstructure on the surface [29,72]. Owing to the reduced water absorption rate of MRFA, the effective water-binder ratio in the paste increases, enabling the full dissolution of Ca, Al, and silicate ions, which enhances the efficiency of the hydration reaction. Residual NS and sodium silicate particles on the MRFA surface can also participate in the alkali-activation reaction of the matrix. The resulting hydration products strengthen the structure of the interfacial transition zone (ITZ) between the MRFA and matrix. These microscopic effects may increase the microhardness of the new mortar near the MRFA surface and interface, which in turn manifests as a significant improvement in the macroscopic compressive strength.

Owing to the residual unreacted silica in the RNMS, the modulus of the activator prepared from it increases, which in turn leads to a slow early strength development of RMRs. This is especially evident in the low replacement ratio groups; for example, the 3-day strength of RMR-1 is 16.2% lower than that of NM, at only 18.6 MPa. This trend of early strength reduction in AASRMs as the modulus of the alkali activator increases is consistent with the results reported in related studies [73]. This is because the higher modulus of the alkali activator weakens the alkaline environment of the paste reaction, hindering the dissolution of the precursors. This suppresses the dissociation of Si-O and Al-O bonds, weakens the polymerization reaction, and ultimately results in a reduced strength [74,75]. However, as the MRFA content increased, this trend significantly improved. RMR-10, which had the highest strength, showed an increase of 50.6% compared to NM and was only 2.7% lower than MR-10. This indicates that the addition of MRFA is the most important factor in enhancing the compressive strength of AASRMs. As the curing time increased, the compressive strength of the RMRs increased significantly, and at 28 d, all exceeded that of the NM, reaching 78.6–95.6% of the strength of the MRs. This shows that as the reaction proceeds, the residual NS and sodium silicate particles on the surface of MRFA react with the matrix to form hydration products, improving the ITZ structure, and in synergy with the modified aggregate, which has better mechanical properties, collectively enhances the overall strength.

### 3.5. Drying Shrinkage

**Figure 9:**
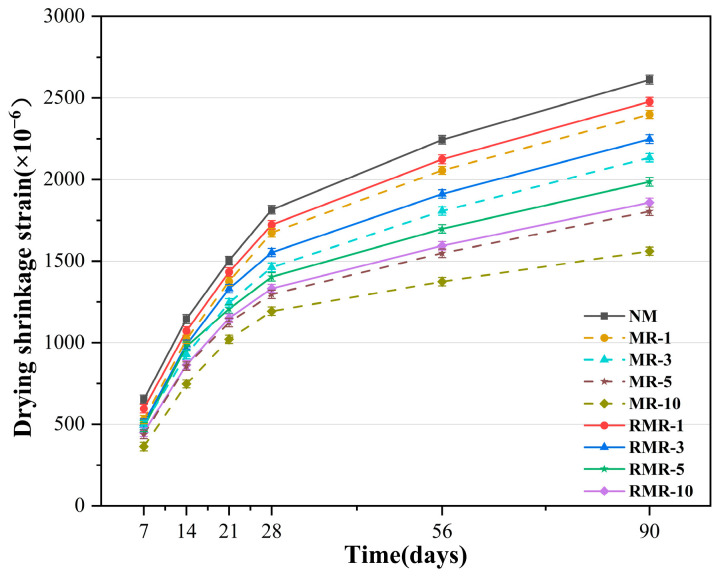
Drying shrinkage of mortars at 28 d.

Figure 9 shows the drying shrinkage strain of the AASRMs mortar. For all specimens, the drying shrinkage strain increased over time. Meanwhile, the drying shrinkage strain developed rapidly within the first 28 d, after which the rate of increase gradually slowed. Among them, NM exhibited the highest drying shrinkage strain of all the specimens, reaching 2612 με at 90 days. This is because RFA has a higher water absorption rate and lower elastic modulus. Higher water absorption leads to more internal moisture evaporation within the AASRMs, thereby increasing the driving force for drying shrinkage [76]. The old adhered cement mortar on the surface weakens the elastic modulus of the RFA, thereby reducing its ability to restrain the mortar’s shrinkage [77]. Therefore, NM, which uses only RFA as an aggregate, exhibited the highest drying shrinkage strain.

The drying shrinkage strain decreased significantly with an increase in MRFA incorporation. Compared with the NM in the control group, the drying shrinkage strain of the MRs was notably reduced at all ages. Among them, the drying shrinkage strain of MR-10 at 90 d is only 1561 με, which is 40.2% lower than that of NM. Generally, drying shrinkage is caused by the migration of water from the pores within the mortar to the environment. Maintaining the humidity of the paste by sealing the pores can effectively reduce the drying shrinkage [78]. Through the physical filling effect of NS and film formation by sodium silicate gel, the NMS modification treatment significantly improved the surface pore structure of RFA, reducing its water absorption and potential drying shrinkage. Meanwhile, the residual NS and sodium silicate particles on the surface of the MRFA can participate in subsequent alkali activation reactions, forming a denser and stronger ITZ. The enhanced ITZ not only improves the compressive strength but also increases the restraint of matrix shrinkage, thereby reducing extra shrinkage or cracking caused by interfacial weakness.

Meanwhile, the drying shrinkage strain of the RMRs also decreased significantly with an increase in the MRFA content. At 90 d, the drying shrinkage strain ranged from 1859 to 2477 με, which is 5.2–28.8% lower than that of NM. However, it is worth noting that as the MRFA content increases, the increase in the drying shrinkage strain of RMRs compared to MRs at the corresponding content at 90 d also shows an upward trend. When only 10% MRFA was added, the drying shrinkage strain of the RMRs was only 3.25% higher than that of the MRs, which is a relatively small difference. However, when MRFA is used entirely, this difference expands to 19.09%. This may indicate that when the MRFA content is low, the changes in modulus and adverse factors such as impurities in the RNMS have a limited impact on the overall drying shrinkage performance, as the properties of the aggregate and the improvement of the ITZ to some extent offset these shortcomings [34]. However, when MRFA completely replaces RFA, the deterioration of alkali-activated raw materials becomes a more prominent factor affecting the final mortar performance, as reflected in its shrinkage property.

### 3.6. Microstructure Analysis

**Figure 10:**
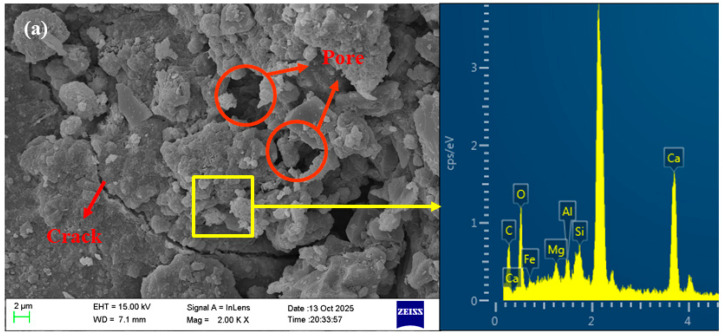

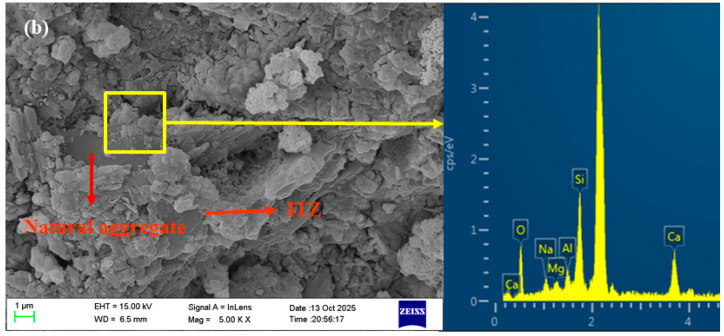
SEM images of surface morphology of (**a**) RFA and (**b**) MRFA with EDS spectrum analysis.

**Figure 11:**
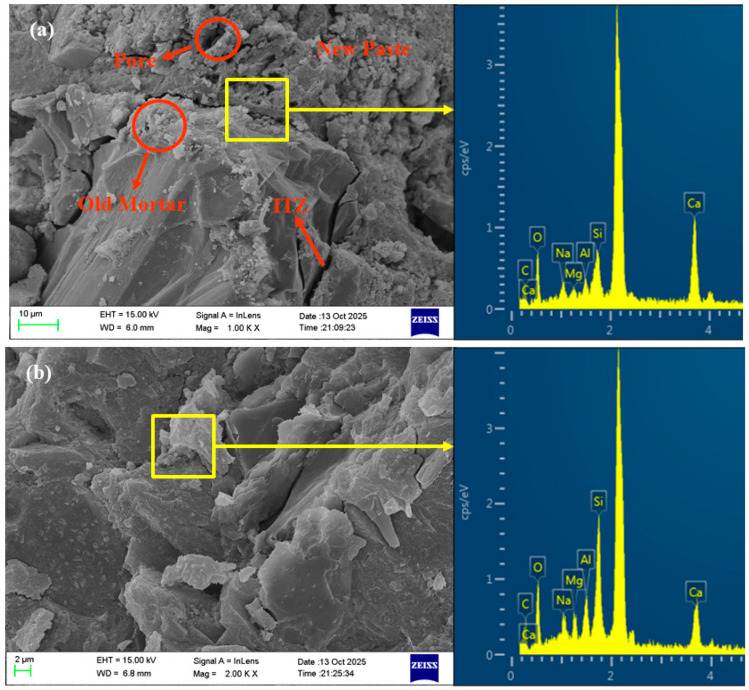
SEM images of ITZ of (**a**) NM and (**b**) MR-10 with EDS spectrum analysis.

**Figure 12:**
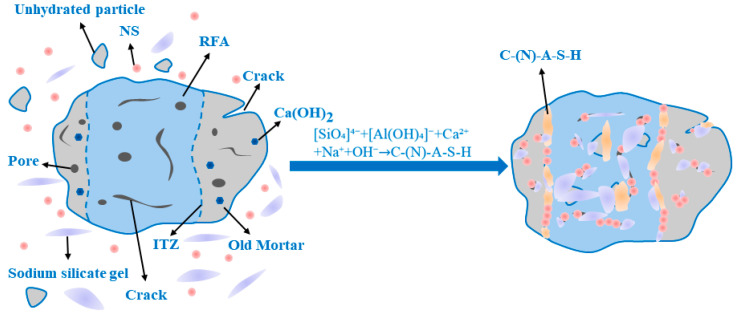
Schematic of NMS modification on the MRFA surface.

Figure 10 and Figure 11 show the SEM images of the 28-day AASRM mortar, and representative areas were analyzed using EDS. Through SEM analysis, the surface morphology of the two types of aggregates and the ITZ of the mortar samples were characterized to reveal the role of NMS modification in improving the mechanical and durability properties of MRFA. As shown in Figure 10a, the surface of untreated RFA is covered with a large amount of residual old mortar, which alters its surface characteristics [27]. Simultaneously, owing to decomposition and crushing during the production process, the RFA surface exhibits high porosity and abundant pores, accompanied by many cracks [79]. EDS analysis of this surface confirmed that its composition was old cement mortar, revealing high levels of Si and Ca, as well as a characteristic S signal from hydration products such as ettringite, while the Na signal was negligible. After strengthening the RFA by soaking modification with NMS, a continuous and relatively homogeneous C-(N)-A-S-H gel film was formed on the aggregate surface, covering most of the aggregate area, as shown in Figure 10b. The EDS spectrum of this new gel layer shows that, compared to untreated RFA, the Na content has increased significantly, and the Si/Ca ratio is very high, confirming the formation of a C-(N)-A-S-H type gel. This is because unhydrated cement particles in the old mortar dissolve and release ions (primarily Ca^2+^ and Al^3+)^, which react with silicate ions from sodium silicate and dissolved NS in the NMS to form C-A-S-H gel. In an alkaline environment, the dissolved silicate ions may also undergo polycondensation to form N-A-S-H gel. Through the physical filling effect of NS and the film-forming effect of sodium silicate gel, the pores and cracks on the RFA surface are repaired, water absorption is reduced, and the effective water-cement ratio of the paste is improved. Simultaneously, loose particles are bonded together and encapsulated by the C-(N)-A-S-H gel to form a dense protective shell, enhancing the stiffness of the aggregate and, in turn, increasing the compressive strength of the AASRMs. Based on the analysis of the SEM images and EDS spectra of the samples, and according to the conceptual model proposed by Liu et al. [34] in the OPC system, the modification mechanism of NMS on the MRFA surface is illustrated in Figure 12.

**Figure 13:**
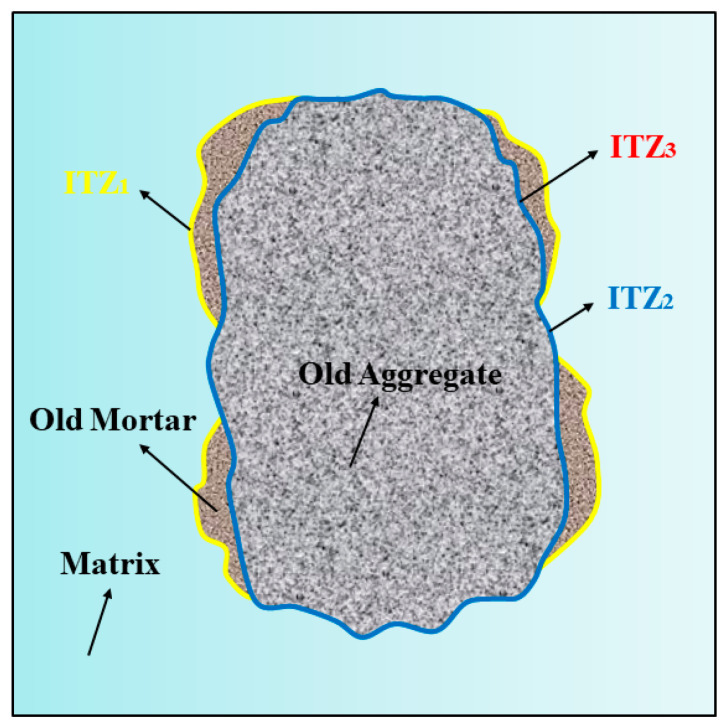
Multiple interface structures of alkali-activated slag mortar with recycled fine aggregates.

**Figure 14:**
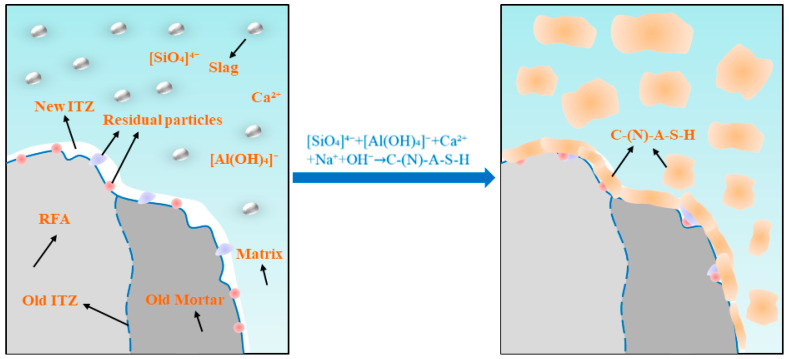
Schematic view of the action mechanism of the MRFA in the interface transition zone.

As shown in Figure 13, based on the model established by Zhang et al. [27], multiple interfacial transition zone (ITZ) structures exist in AASRMs owing to the adhered old mortar on the surface of the RFA: (a) ITZ1 exists between the old mortar and the matrix; (b) ITZ2 exists between the old aggregate and the matrix; and (c) ITZ3 exists between the old aggregate and the old mortar. Previous research has concluded that, compared to the matrix paste, the more porous ITZ is typically considered the weakest link between the aggregate and matrix. In particular, when subjected to external loads, the porous ITZ often provides an easier path for crack propagation. In Figure 11a, the ITZ between the unmodified RFA and the matrix is very distinct, and clear cracks are observed at the interface between the aggregate and the matrix. This is because the high porosity and low water content of RFA cause it to absorb water from the surrounding matrix after being mixed into the paste. This leads to water loss in the ITZ, leaving behind a large number of pores and generating cracks [9]. These cracks weaken the ITZ structure, thereby reducing the mechanical and durability performance of the mortar. However, as observed in Figure 11b, the MRFA and the surrounding matrix were tightly bonded and filled with C-(N)-A-S-H gel, with no obvious cracks observed. Owing to the filling effect of NS and the film-forming effect of sodium silicate gel, the pores and cracks on the RFA surface were repaired, enhancing the aggregate strength while reducing water absorption. This process reduces the amount of water absorbed by the MRFA during mixing in the paste, thereby suppressing water loss at the interfacial transition zone (ITZ) and effectively preventing the formation of pores and cracks. As shown in Figure 14, the reaction between the MRFA surface and the matrix can be intuitively understood, thereby optimizing the ITZ structure. Because unreacted NS and sodium silicate particles remain on the surface of MRFA after NMS soaking modification, they can serve as nucleation sites and participate in the alkali-activation reaction of the matrix. This promoted the uniform growth of the gel in the ITZ, filling the micropores and optimizing the pore structure. EDS analysis of the ITZ region in the MR-10 sample confirmed a significant enrichment of Si and Na in this area, with the Na content being the highest among all analysis points. The pre-formed gel layer on the MRFA surface forms a stronger chemical and physical bond with the alkali-activated slag paste, effectively bridging the aggregate and paste, thus reinforcing the ITZ and transforming it from a traditional weak link. As the incorporation of MRFA increased, more ITZs were strengthened, which then translated into gradual improvements in the mechanical and durability performance at the macro scale.

### 3.7. Economical and Environmental Analysis

While the integration of MRFA enhances the performance of AASRMs, a comprehensive assessment of its economic and environmental impacts is crucial. It is worth noting that the recycling of RNMS can reduce the waste of raw materials to some extent. Therefore, a five-dimensional assessment of AASRMs was conducted, considering embodied carbon, mechanical properties, material cost, ecological efficiency, and cost-effectiveness, to evaluate their impacts [80]. Table 4 lists the estimated carbon content and raw material costs of the AASRMs, including the entire process from raw material extraction and processing to the completion of the mortar production. As RNMS is a waste material, its cost and embodied carbon are considered to be zero. The results for environmental value can be calculated as CO_2_ emissions using Equations (2) and (3) is used for economic assessment [81].

(2) $$Eco-efficiency=\frac{Compressive\;strength\;at\;28\;d}{Embodied\;carbon}$$

(3) $$Cost-benefit=\frac{Compressive\;strength\;at\;28\;d}{Total\;material\;prices}$$

**Figure 15:**
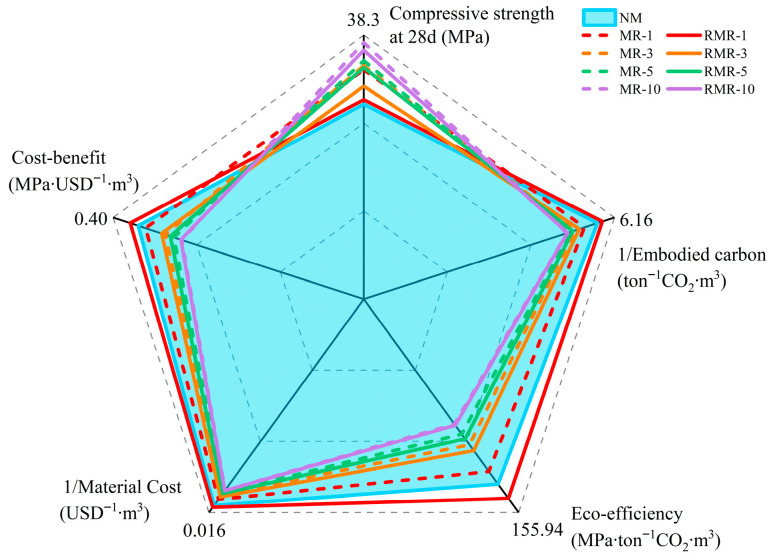
Five-dimensionality assessment considering compressive strength, embodied carbon, eco-efficiency, material cost, and cost–benefit of AASRMs with varying MRFA incorporation and RNMS utilization.

Figure 15 demonstrates and evaluates the eco-efficiency, material cost, embodied carbon, and cost-effectiveness of AASRMs for different mortar mix ratios. As the amount of MRFA increases, both the material cost and embodied carbon increase, which in turn leads to a decline in eco-efficiency and cost-effectiveness. This is because modifying RFA consumes more NS and water glass solution, which, while enhancing mechanical and durability performance, reduces economic and environmental advantages. However, by using RNMS as the raw material for alkali activators, eco-efficiency and cost-effectiveness can be effectively improved at lower replacement rates, and RMR-1 significantly outperformed the blank NM group. Simultaneously, RMRs have better sustainability and higher cost-effectiveness than MRs at each corresponding replacement rate. However, as the MRFA content increased, both the cost and embodied carbon of the RMR group increased. This is because commercial sodium silicate and NS are consumed during the modification process. Therefore, when MRFA is used in small amounts, utilizing RNMS as an alkali activator can ensure economic and environmental benefits.

## 4. Conclusions

This study aims to modify RFA using a mixed solution of 2 wt% NS and 15 wt% sodium silicate and explore the feasibility of using RNMS as a source material for alkali activators. The workability, phase composition, capillary water absorption, mechanical properties, drying shrinkage, and microstructure of AASRMs with different MRFA contents were studied, and their economic and environmental impacts were analyzed. The main conclusions are as follows:

(1) Immersion modification of RFA using NMS significantly improved the overall performance of AASRMs. As the amount of MRFA added increased, the mortar’s workability improved significantly, while capillary water absorption and drying shrinkage were effectively suppressed, and the compressive strength was significantly enhanced. In particular, MR-10 achieved a 28-day compressive strength as high as 38.3 MPa, with capillary water absorption height and drying shrinkage strain reduced by 49.5% and 40.2%, respectively, compared to the control NM. This indicates that the incorporation of MRFA effectively remedied the defects of RFA, thereby improving the mechanical and durability properties of mortar.
(2) The pores and cracks on the surface of the RFA were repaired through the filling effect of NS and the film-forming action of sodium silicate gel. This reduces the water absorption rate of MRFA and encapsulates loose microparticles to form a dense protective shell, thereby enhancing the stiffness of the aggregate. NS and sodium silicate can be transported into the RFA through microcracks, where they react with Ca^2+^ and Al^3+^ to form C-(N)-A-S-H gel, thereby strengthening the microstructure of the ITZ. Simultaneously, residual NS and sodium silicate particles on the surface of MRFA serve as nucleation sites, promoting the uniform growth and densification of C-(N)-A-S-H gel in the new ITZ, thus reinforcing the previously weak ITZ. This microstructure optimization effectively inhibited capillary water absorption and drying shrinkage and significantly improved the mechanical properties of the AASRMs.
(3) Using RNMS as an alkali activator raw material can ensure performance advantages at different MRFA dosages. Although RMRs exhibit slightly slower early strength development and have slightly higher capillary water absorption and drying shrinkage strain than MRs with the same MRFA content, their performance is still far superior to that of the blank NM group, and their fluidity is basically consistent with that of MRs. This indicates that RNMS, as an alkali activator raw material, can effectively promote the alkali activation reaction of the matrix and maintain the excellent performance of AASRMs with MRFA incorporation.
(4) From the perspectives of economics and the environment, although the incorporation of MRFA increases material costs and embodied carbon, the recycling and reuse of NMS significantly improve the eco-efficiency and cost-effectiveness of AASRMs. At low replacement rates, the overall benefits of RMR-1 surpassed those of the blank NM group, with the embodied carbon reduced by 11.9%. With the same amount of MRFA added, RMRs demonstrated better sustainability and higher cost-effectiveness than MRs. This confirms that the simultaneous use of RNMS and MRFA can enhance the performance of AASRMs while balancing economic and environmental benefits.
(5) Based on the findings of this study, several key directions for future research are recommended to advance the field. First, it is essential to conduct a comprehensive investigation of the long-term durability of AASRMs prepared with recycled activators, including their performance under freeze–thaw cycles, sulfate attack, and long-term drying shrinkage. To deepen our understanding of these mechanisms, future studies should incorporate quantitative microstructural analyses. Although this study qualitatively links microstructural improvements to performance, techniques such as quantitative image analysis of backscattered electron (BSE-SEM) micrographs can be used to establish direct numerical correlations between key microstructural indicators (such as porosity or gel fraction from image analysis) and observed macroscopic performance. Second, the applicability of this NMS pretreatment method can be explored in other AAM systems, such as low-calcium fly ash-based geopolymers, in which the interaction mechanisms may differ. Finally, detailed techno-economic analyses and comprehensive life cycle assessments (LCA) will provide valuable data for quantifying the actual economic and environmental benefits of this integrated “modification–recycling” approach, paving the way for its potential industrial application.

## Figures and Tables

**Table 1:** Chemical composition of GGBS (%).

CaO	SiO_2_	Al_2_O_3_	MgO	Fe_2_O_3_	SO_3_	K_2_O	Other
45.06	26.71	16.34	7.80	0.52	2.32	0.36	0.89

**Table 2:** Physical and mechanical properties of different aggregates.

Type	Bulk Density (kg/m^3^)	Apparent Density (kg/m^3^)	Fineness Modulus	Maximum Particle Size (mm)	Water Absorption (%)
RFA	1320	2385	2.69	2.5	8.86
MRFA	1297	2463	2.81	2.5	5.75

**Table 3:** Mix proportion design of different mortars (kg/m^3^).

Group	GGBS	Aggregate		Waterglass Solution	RNMS	NaOH	Additional Water
		RFA	MRFA				
NM	312	1320	—	69.2	—	17.5	234.9
MR-1	312	1188	132	69.2	—	17.5	234.9
MR-3	312	924	396	69.2	—	17.5	234.9
MR-5	312	660	660	69.2	—	17.5	234.9
MR-10	312	—	1320	69.2	—	17.5	234.9
RMR-1	312	1188	132	—	138.3	17.5	165.8
RMR-3	312	924	396	—	138.3	17.5	165.8
RMR-5	312	660	660	—	138.3	17.5	165.8
RMR-10	312	—	1320	—	138.3	17.5	165.8

**Table 4:** Embodied carbon and material costs of raw materials.

Materials	Embodied Carbon (Metric Ton eq. CO_2_/Metric Ton)	Material Costs (USD/Metric Ton)
RFA	0.012 [82]	8.64
GGBS	0.019 [83]	36.8
Sodium silicate solution	1.860 [84]	602.9
NaOH	1.915 [83]	269.6
Tap water	0.001 [83]	0.5
Nano-SiO_2_	0.00084 [85]	473.6

## Data Availability

The original contributions presented in this study are included in the article. Further inquiries can be directed to the corresponding author.

## Abbreviations

The following abbreviations are used in this manuscript:

RFA	Recycled fine aggregate
MRFA	Modified recycled fine aggregate
NMS	Nano-SiO_2_–sodium silicate mixed solution
RNMS	Recycled nano-SiO_2_–sodium silicate mixed solution
NS	Nano-silica
AASRM	Alkali-activated slag recycled fine aggregate mortar
C&DW	Construction and demolition waste
ITZ	Interfacial transition zone
NA	Natural aggregate
RA	Recycled aggregate
CH	Calcium hydroxide
C-S-H	Calcium–silicate–hydrate
C-(N)-A-S-H	Calcium–(sodium)–aluminate–silicate–hydrate
OPC	Ordinary Portland Cement
GGBS	Ground granulated blast-furnace slag
AAM	Alkali-activated material
SEM	Scanning electron microscopy
XRD	X-ray diffraction
XRF	X-ray fluorescence
Ms	Modulus
